# Monitoring the Process of Endostar-Induced Tumor Vascular Normalization by Non-contrast Intravoxel Incoherent Motion Diffusion-Weighted MRI

**DOI:** 10.3389/fonc.2018.00524

**Published:** 2018-11-13

**Authors:** Jing-hua Pan, Shengbin Zhu, Jinlian Huang, Jianye Liang, Dong Zhang, Xiaoxu Zhao, Hui Ding, Li Qin, Changzheng Shi, Liangping Luo, Yunlong Pan

**Affiliations:** ^1^Department of General Surgery, The First Affiliated Hospital of Jinan University, Guangzhou, China; ^2^Medical Imaging Center, The First Affiliated Hospital of Jinan University, Guangzhou, China; ^3^Department of Histology and Embryology, Medical School of Jinan University, Guangzhou, China

**Keywords:** IVIM-DWI, MRI, tumor vascular normalization, Endostar, perfusion

## Abstract

Tumor vascular normalization has been proposed as a new concept in anti-tumor angiogenesis, and the normalization window is considered as an opportunity to increase the effect of chemoradiotherapy. However, there is still a lack of a non-invasive method for monitoring the process of tumor vascular normalization. Intravoxel incoherent motion diffusion-weighted magnetic resonance imaging (IVIM DW-MRI) is an emerging approach which can effectively assess microperfusion in tumors, without the need for exogenous contrast agents. However, its role in monitoring tumor vascular normalization still needs further study. In this study, we established a tumor vascular normalization model of CT26 colon-carcinoma-bearing mice by means of Endostar treatment. We then employed IVIM DW-MRI and immunofluorescence to detect the process of tumor vascular normalization at different times after treatment. We found that the D^*^ values of the Endostar group were significantly higher than those of the control group on days 4, 6, 8, and 10 after treatment, and the f values of the Endostar group were significantly higher than those of the control group on days 6 and 8. Furthermore, we confirmed through analysis of histologic parameters that Endostar treatment induced the CT26 tumor vascular normalization window starting from day 4 after treatment, and this window lasted for 6 days. Moreover, we found that D^*^ and f values were well correlated with pericyte coverage (*r* = 0.469 and 0.504, respectively; *P* < 0.001, both) and relative perfusion (*r* = 0.424 and 0.457, respectively; *P* < 0.001, both). Taken together, our findings suggest that IVIM DW-MRI has the potential to serve as a non-invasive approach for monitoring Endostar-induced tumor vascular normalization.

## Introduction

Anti-angiogenesis therapy is widely used in the clinical treatment of various solid tumors. However, clinical studies have shown that anti-angiogenesis monotherapy does not significantly improve tumor response rate or overall survival (1). On the basis of the concept of anti-tumor angiogenesis, Professor Jain put forward the theory of “normalization of tumor blood vessels” in 2001 (2). The theory states that, after the process of appropriate anti-angiogenic therapy—by equilibrating anti-angiogenic factors and angiogenic stimulating factors, which promote vascular maturation and pericyte coverage—the normalized vascular structures in tumors will contain well-structured and regular basement membranes, which reduce the leakage of blood and distortion of the vascular network in solid tumors (3). Furthermore, a normalized tumor vascular network with good vascular structure contributes toward increasing intratumoral blood perfusion and improving the tumor hypoxia environment. Several studies (4–6) have shown that tumor vascular normalization induced by vascular endothelial growth factor (VEGF) signaling inhibitors, such as bevacizumab and Endostar (recombinant human endostatin), significantly improves the outcomes of chemotherapy and radiotherapy. Therefore, the concept of tumor vascular normalization offers new opportunities in tumor anti-angiogenesis therapy.

Anti-angiogenesis therapy for vascular normalization involves achieving a “normalization window,” which is a time period beginning with the appearance of a normalized vascular phenotype and ending when the features of normalization are lost (7). Since the phenotype of normalizing tumor blood vessels is transient, the duration of the normalization window is relatively narrow and depends on the dosage of VEGF inhibitors and tumor type (8). Several preclinical studies have demonstrated the features of vascular normalization, including the normalized structure and function of the tumor vascular network (9, 10). The structural features of normalized tumor vessels include reduced vessel diameter, increased pericyte coverage or proximity, and normalized basement membrane, and their functional features include improved oxygenation, reduced permeability or interstitial fluid pressure, and improved delivery of drugs or systemically administered molecules (11). These features of tumor vascular normalization are mainly monitored by pathological evaluation methods such as immunohistochemical analysis and immunofluorescence. However, these invasive methods greatly limit their further application for monitoring the process of tumor vascular normalization in clinical settings. Although these pathological methods help identify some tumor vascular normalization features at particular points in time, it is still challenging to identify the beginning and end points of the normalization window of tumor vascular normalization in patients.

Non-invasive magnetic resonance imaging (MRI) plays an important role in the evaluation of antivascular therapies and has been widely used in clinical settings. A previous study reported that dynamic contrast-enhanced (DCE)-MRI is frequently used for monitoring anti-angiogenic and vascular disrupting compounds (12). Although DCE-MRI signals can reflect the physical and physiological characteristics of a target organ or tissue, their enhancement will be affected by the concentration of the contrast agent; moreover, the use of contrast agents increases the risk of adverse events such as allergy-like reactions, nephrotoxicity, extravasation, and nephrogenic systemic fibrosis (13). In addition, although diffusion-weighted imaging (DWI) and ^19^F MRI have been used for monitoring changes in tumor cellularity and mapping oxygenation by imaging lipid relaxation enhancement (14, 15), these methods cannot be used frequently because of the risk of deposition of contrast agents. Consequently, with repeated use, it is difficult to monitor the process of vascular normalization through these methods.

Intravoxel incoherent motion (IVIM) DWI is an imaging method used for describing the microscopic motion of voxels. This microscopic movement of organisms includes the diffusion of water molecules and microcirculation of blood (16). Not only can IVIM-DWI provide quantitative parameters for tissue diffusion, but it can also reflect tissue microperfusion. In comparison to the traditional DWI mathematical model, IVIM-DWI uses a biexponential model to detect the dispersion of water molecules in tissues, which can help better describe this complex signal attenuation *in vivo* (17). Moreover, IVIM DW-MRI has been used for assessment of tumor perfusion, and the results have indicated that the blood pseudo-diffusion coefficient (D^*^) and perfusion fraction (f) can satisfactorily reflect tumor angiogenesis and microvessel density (18, 19). A further study has shown that IVIM DW-MRI can be used to predict the efficacy of antivascular therapies without the need for contrast-medium injection (20).

Anti-angiogenesis therapy is an attractive approach for treatment of metastatic colorectal cancer. Recombinant human endostatin (Endostar) is an endogenous inhibitor of angiogenesis, and previous studies have reported that Endostar can efficiently block angiogenesis and normalize the tumor vasculature in colorectal cancer (21). The findings of histologic assessment of the vascular structure have revealed that the tumor vascular normalization window induced by Endostar mainly starts on days 3 or 4 after treatment and is maintained for ~4–8 days in a mouse model (22). In the present study, we used Endostar to create a vascular normalization window in a colorectal cancer model and investigated whether IVIM DW-MRI can be used as a novel method for monitoring the process of tumor vascular normalization. Further, we also studied the correlation between the perfusion-related IVIM-DWI parameters and pathological parameters of normalized tumor vessels.

## Materials and methods

### Cell line and animals

The CT26 murine colon carcinoma cell line was purchased from the Type Culture Collection of the Chinese Academy of Sciences (Shanghai, China). It was cultured in Dulbecco's Modified Eagle's Medium (DMEM) containing 10% fetal bovine serum (Thermo Fisher Scientific, Waltham, MA, USA) and 1% penicillin/streptomycin at 37°C in 5% CO_2_.

### Animal model

**Figure 1:**
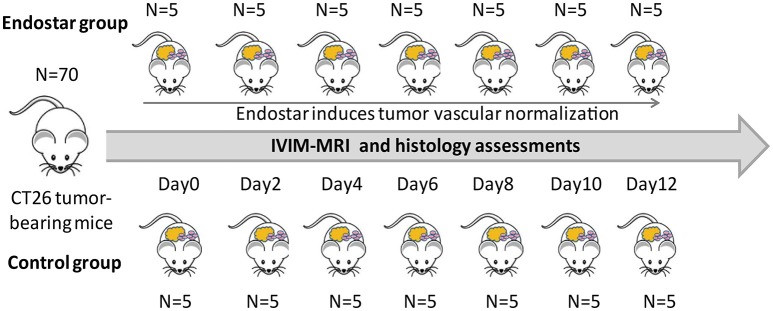
Schematic diagram of the design and treatment process of the study. A total of 70 Balb/c were randomly divided into the Endostar and control groups (*n* = 35, each). Five mice in each group were randomly selected for IVIM-MRI and sacrifice for histologic analysis at each time point from days 0 to 12. IVIM, intravoxel incoherent motion; MRI, magnetic resonance imaging.

The procedures for animal experiments in mice were approved by the Laboratory Animal Ethics Committee of Jinan University. The experiments were strictly conducted in accordance with the relevant recommendations. A total of 70 female Balb/c mice (6–8 weeks old) were purchased from Beijing HFK Bioscience Co., LTD (Beijing, China) and kept in specific pathogen-free conditions. For creating CT26 tumor-bearing mice, CT26 cells (1 × 10^7^) were subcutaneously injected into the right flank of the mice. Then, tumor size was monitored using calipers and calculated using the following formula: volume = (length × width^2^) × 0.523. After injection for 10 days, the mice were randomly divided into two groups: the Endostar and control groups. Mice in the Endostar group received 5 mg/kg Endostar via intravenous injection every day before detection, while those in the control group received the same volume of 0.9% saline. Mice that had developed tumors were randomly selected for MRI and structural and functional vascular analysis before treatment (0 d) and on days 2, 4, 6, 8, 10, and 12 after treatment (five mice from each group, each day; Figure 1). Each mouse underwent MRI only once before sacrifice and tumor excision.

### MRI investigation

All MRI investigations were conducted using a 1.5-T Signa HDxt superconductor clinical MR system (GE Medical System) equipped with a human eight-channel wrist coil and a maximum gradient strength of 45 mT/m. The animals were anesthetized by intraperitoneal injection of 0.3% pentobarbital and imaged in the supine position. All IVIM-DW MR images were acquired using a single-shot, echo-planar imaging pulse sequence with following imaging parameters: repetition time, 4,000 ms; echo time, 91.8 ms; slice thickness, 2.0 mm; slice gap, 0.2 mm; matrix size, 128 × 96; field of view, 10 × 7 cm^2^; small delta, 24.2 ms; big delta, 30.2 ms; and flip angle, 90°. The diffusion gradients applied in three orthogonal directions included 13 b values: 0, 25, 50, 75,100, 150, 200, 400, 600, 800, 1,000, 1,200, and 1,500 s/mm^2^. Fat suppression was achieved using the chemical shift-selective saturation technique. The acquisition time for each DW image was 7 min 31 s.

### Image post-processing and analysis

For the IVIM-DW images, quantitative analysis and measurement of variables was performed prospectively by two experienced radiologists with more than 5 years of experience. As previously described (20), all IVIM sequence data were transferred to a dedicated post-processing workstation (AW4.5, GE Healthcare, Little Chalfont, Buckinghamshire,UK), and quantitative analysis was performed using the IVIM software package. The IVIM-DWI data were analyzed with the Functool MADC program, using a biexponential model defined by *SI/SI*_0_ = *(1–f)*·*exp(–bD)+ f*·exp(–bD^*^), where SI_0_ is the mean signal intensity of the region of interest (ROI) for a b value of 0, and SI is the signal intensity for higher b values. The evaluated IVIM parameters included the slow apparent diffusion coefficient (ADC) (D), the fast ADC (D^*^), and the fraction of the fast ADC (f). While D and D^*^ represent the true diffusion and pseudo-diffusion coefficients, respectively, f represents the perfusion fraction. For assessment of IVIM parameters, ROIs were manually drawn by outlining the tumor border on the T2-weighted image that showed the largest cross-sectional tumor area.

### Immunofluorescence and analysis of vascular perfusion

On days 0, 2, 4, 6, 8, 10, and 12 after treatment, five mice in each group were randomly selected for analysis of vessel density, pericyte coverage, and vascular perfusion by immunofluorescence (Figure 1). In brief, 10 mg/kg fluorescein isothiocyanate (FITC)-conjugated lectin (Sigma-Aldrich, USA) was injected intravenously for 20 min before tumor excision. For analysis of vessel density and pericyte coverage, tumor tissues were fixed in 4% paraformaldehyde for 24 h and embedded in paraffin. Then, the tissue specimens were sectioned, dewaxed in xylene, and rehydrated using a graded alcohol series. Antigen retrieval was performed in citric acid buffer (pH 6.0). The sections were blocked with 2% normal goat serum for 1 h and then stained with the following primary antibodies: Anti-CD31 (cluster of differentiation 31) antibody (1:500; Abcam, Cambridge, MA, USA) for the endothelium and α-SMA (smooth muscle actin) antibody (1:100; Proteintech, Chicago, IL, USA) for pericytes. The specimens were then washed and incubated with rhodamine-conjugated goat anti-rat immunoglobulin G (IgG; H+L; 1:50; Proteintech) or FITC-conjugated goat anti-rabbit IgG (1:200; Santa Cruz, CA, USA) for 40 min. Vessel density was determined by the extent of CD31+ area per scope ( × 200) using the Image J software. Pericyte coverage was determined as the percentage of α-SMA+ area/CD31+ area. For analysis of vascular perfusion, tumor tissues were frozen in optimal cutting temperature compound (Sakura Finetek, Torrance, CA, USA) and stored at −80°C. Cryosections with a thickness of 20 μm were fixed in cold acetone and rehydrated in phosphate-buffered saline. Microvessels were stained with an anti-CD31 antibody (1:500; Abcam), and relative perfusion was calculated as the ratio of lectin+ area/CD31+ area using the Image J software.

### Statistical analysis

For quantitative analysis, the distribution of the extracted IVIM parameters is shown as median and/or mean value with standard deviation (SD) and range. The histologic parameters and the D, D^*^, and f values of IVIM DW-MRI were tested for Gaussian distribution using the Kolmogorov–Smirnov test. Significant differences in IVIM parameters between the Endostar and control groups were tested using a two-tailed *t*-test. A probability level of *P* < 0.05 was considered statistically significant. Intergroup differences in tumor volume at different time points were analyzed by repeated measures analysis of variance. Correlations between IVIM-derived parameters and histologic parameters were investigated by calculating Spearman's correlation coefficients using GraphPad Prism (version 5.0; GraphPad Software, La Jolla, CA, USA; ^*^P < 0.05, ^**^P < 0.01, and ^***^P < 0.001).

## Results

### Effect of endostar on colon tumor growth

**Figure 2:**
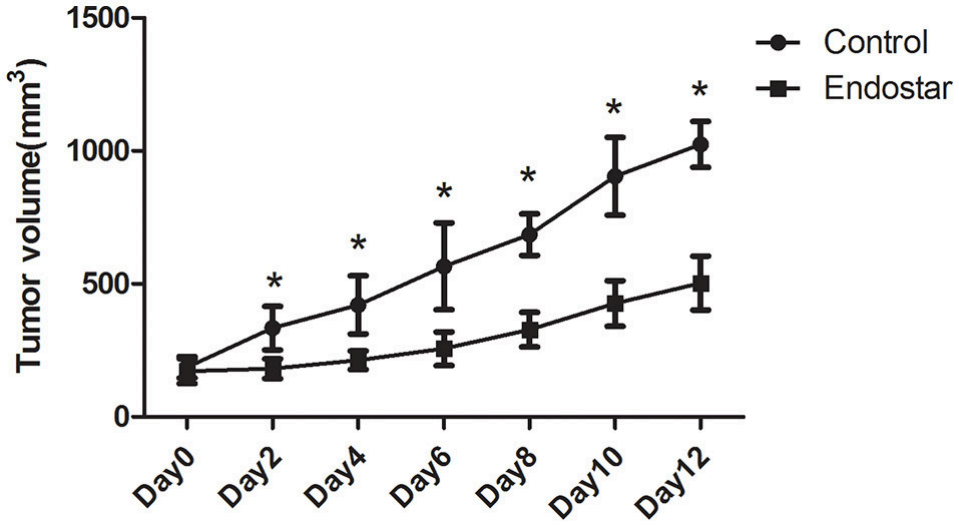
Mouse CT26 tumor growth is hindered by Endostar treatment. Tumor volumes in the Endostar group were significantly lower than those in the control group on days 2, 4, 6, 8, 10, and 12 after treatment. *P < 0.05.

First, we evaluated the effect of Endostar on CT26 tumor growth. As shown in Figure 2, after Endostar treatment for 2–12 days, the tumor volumes in the Endostar group were significantly lower than those in the control group. This suggested that intravenous injection of Endostar at 5 mg/kg/d produced anti-tumor effects in CT26 tumor-bearing mice.

### Assessment of vascular normalization window by analysis of IVIM parameters

**Figure 3:**
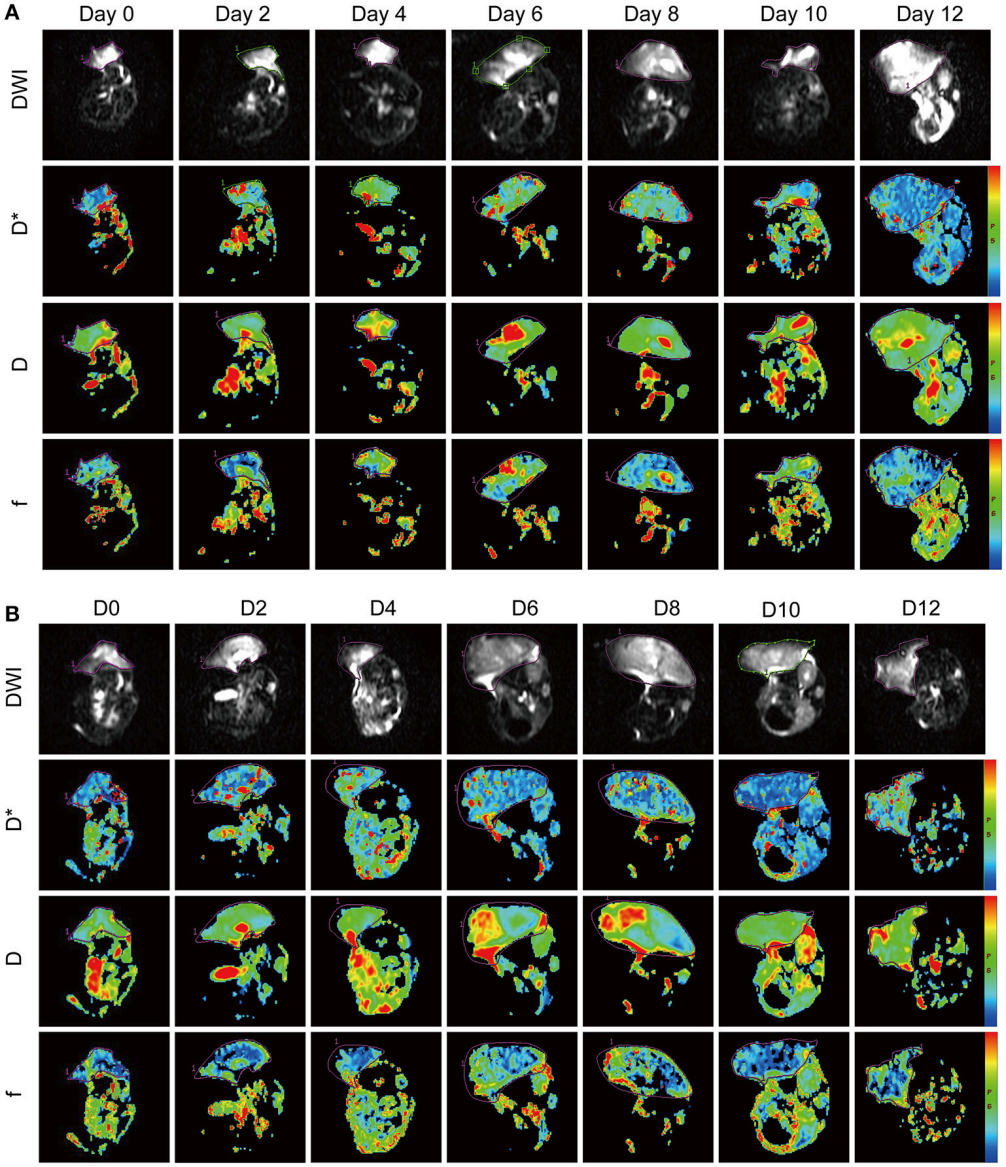
IVIM-DWI MRI features of the CT26 colon cancer mouse model in the Endostar **(A)** and control **(B)** groups at different time points after treatment. The panel shows diffusion-weighted images (DWI) with b = 0 s/mm2 and parametric maps (D*, D, and f) of representative Endostar- and saline-treated tumors on days 0, 2, 4, 6, 8, 10, and 12 after treatment. IVIM, intravoxel incoherent motion; MRI, magnetic resonance imaging.

**Figure 4:**
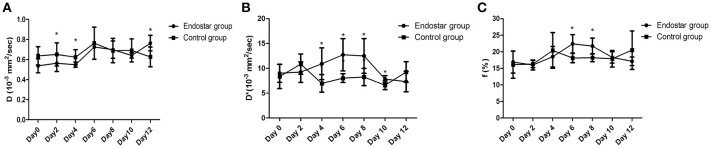
Comparison of D, D*, and f values between the Endostar and control groups at different time points after treatment. **(A)** The differences in D value between the two groups were significant on days 2, 4, and 12 after treatment (*P < 0.05). **(B)** The D*values of the Endostar group were significantly higher than those of the control group on days 4, 6, 8, and 10 after treatment (*P < 0.05). **(C)** On days 6 and 8 after treatment, the f values of the Endostar group were significantly higher than those of the control group (*P < 0.05).

The IVIM parameters evaluated in this study included D, D^*^, and f values. It is still unclear whether these IVIM-derived parameters can be used as a novel measure for monitoring the process of tumor vascular normalization. Therefore, we monitored the IVIM parameters of CT26 tumor-bearing mice in the Endostar and control groups for 0–12 days after treatment. Figure 3 shows representative IVIM images—including parametric maps of DWI, D, D^*^, and f values—of the same tumor sections. Table 1 presents a summary of the serial measurements of the IVIM DW-MRI parameters of CT26 tumor xenografts in the Endostar and control groups at different time points after treatment. In comparison to the D value of the control group, that of the Endostar group was significantly lower on days 2 and 4 after treatment (*P* < 0.05) and significantly higher on day 12 (*P* < 0.05; Figure 4A). There was no significant difference in D^*^ value between the two groups on days 0, 2, and 12 after treatment. Nevertheless, the D^*^ value of the Endostar group gradually increased after day 2 and decreased after day 8 and was significantly higher than that of the control group on days 4, 6, 8, and 10 after treatment (*P* < 0.05; Figure 4B). The f value of the Endostar group exhibited a similar trend as its D^*^ value. The f value of the Endostar group was significantly higher than that of the control group on days 6 and 8 (*P* < 0.05; Figure 4C).

**Table 1:** IVIM-MRI parameters of CT26 xenografts in the Endostar group and control group.

**IVIM-MRI parameter**	**Time(d)**	**Endostar group**	**Control group**	***P***
D (10^−3^ mm^2^/s)	0	0.538 ± 0.070	0.637 ± 0.091	0.090
	2	0.564 ± 0.082	0.652 ± 0.115	**0.037**
	4	0.549 ± 0.026	0.624 ± 0.075	**0.043**
	6	0.726 ± 0.015	0.764 ± 0.160	0.611
	8	0.699 ± 0.086	0.690 ± 0.121	0.896
	10	0.641 ± 0.026	0.691 ± 0.115	0.371
	12	0.764 ± 0.078	0.627 ± 0.098	**0.040**
D*(10^−3^ mm^2^/s)	0	9.010 ± 1.824	8.348 ± 2.423	0.639
	2	9.238 ± 2.072	10.840 ± 2.040	0.253
	4	10.910 ± 3.191	6.964 ± 1.759	**0.042**
	6	12.720 ± 3.238	8.032 ± 0.871	**0.014**
	8	12.490 ± 3.495	8.246 ± 1.737	**0.041**
	10	7.824 ± 0.697	6.590 ± 0.883	**0.040**
	12	7.330 ± 2.047	9.264 ± 2.075	0.176
f (%)	0	16.9 ± 3.3	16.1 ± 4.1	0.763
	2	16.0 ± 1.5	16.3 ± 1.1	0.700
	4	18.5 ± 3.1	20.4 ± 5.4	0.526
	6	22.4 ± 2.8	18.1 ± 1.4	**0.015**
	8	21.7 ± 2.4	18.2 ± 1.2	**0.020**
	10	18.3 ± 1.7	17.9 ± 2.5	0.785
	12	17.1 ± 1.3	20.5 ± 5.8	0.239

*Bold values indicate P < 0.05*.

### Assessment of vascular normalization window by histologic analysis of vascular structure

**Figure 5:**
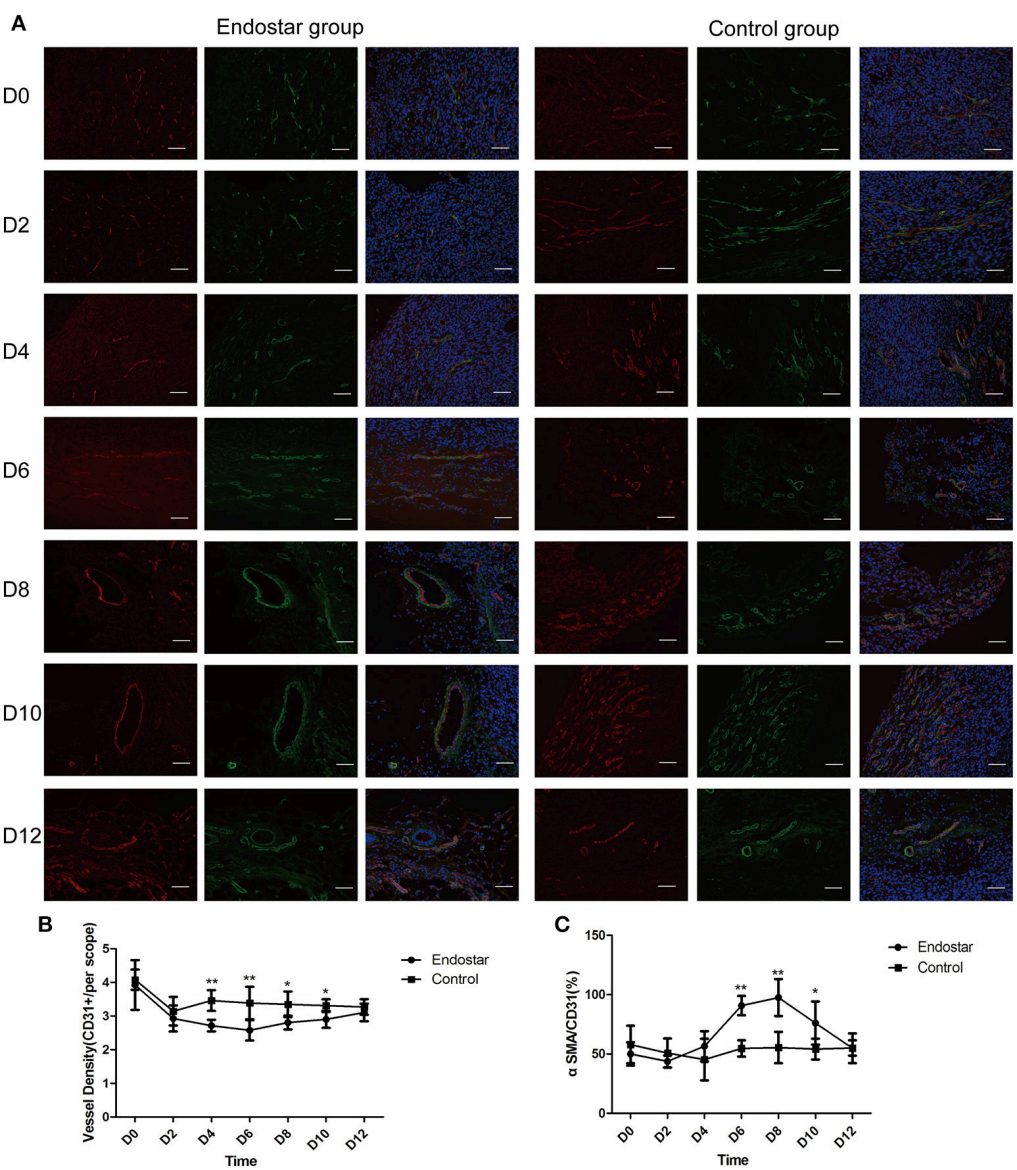
Evaluation of the vascular normalization window by histologic analysis of the vascular structure on days 0, 2, 4, 6, 8, 10, and 12 after treatment. **(A)** CD31 (red), α-SMA (green), and DAPI (blue) staining of representative tumor sections of the Endostar and control groups at different time points. Scale bars: 200 μm. **(B)** Comparison of changes in vessel density (CD31+ area) between the Endostar and control groups at each time point (*P < 0.05; **P < 0.01). **(C)** Comparison of changes in pericyte coverage (CD31+ area/α-SMA+ area) between the Endostar and control groups at each time point (*P < 0.05; **P < 0.01). CD31, cluster of differentiation 31; SMA, smooth muscle actin; DAPI, 4′,6-diamidino-2-phenylindole.

Vessel density and vascular structure are crucial parameters for evaluating the effect of anti-angiogenic treatment. Therefore, we evaluated the vascular normalization window by histologically analyzing the tumor vascular structure. Using CD31 as a vascular-endothelium marker and α-SMA as pericyte marker, we compared the vessel densities and pericyte coverage of the Endostar and control groups from days 0 to 12 after treatment. As shown in Figure 5A, the pericyte coverage in the Endostar group showed an obvious enhancement for 4 days after treatment and a gradual decrease after day 8. The vessel density of the Endostar group was significantly lower than that of the control group on days 4, 6, 8, and 10 after treatment (*P* < 0.05; Figure 5B). The pericyte coverage in the Endostar group was significantly higher than that in the control group on days 6, 8, and 10 after treatment (*P* < 0.05; Figure 5C). These results revealed that Endostar treatment inhibited vascular endothelial growth and enhanced pericyte coverage, which led to normalization of the tumor vascular structure. On the basis of the trends in variation and significant differences in the histologic findings of vascular structure between the two groups, we deduced that Endostar treatment induced the CT26 tumor normalization window starting from day 6 and lasting up to day 10 after treatment.

### Assessment of vascular normalization window by histologic analysis of vascular perfusion

**Figure 6:**
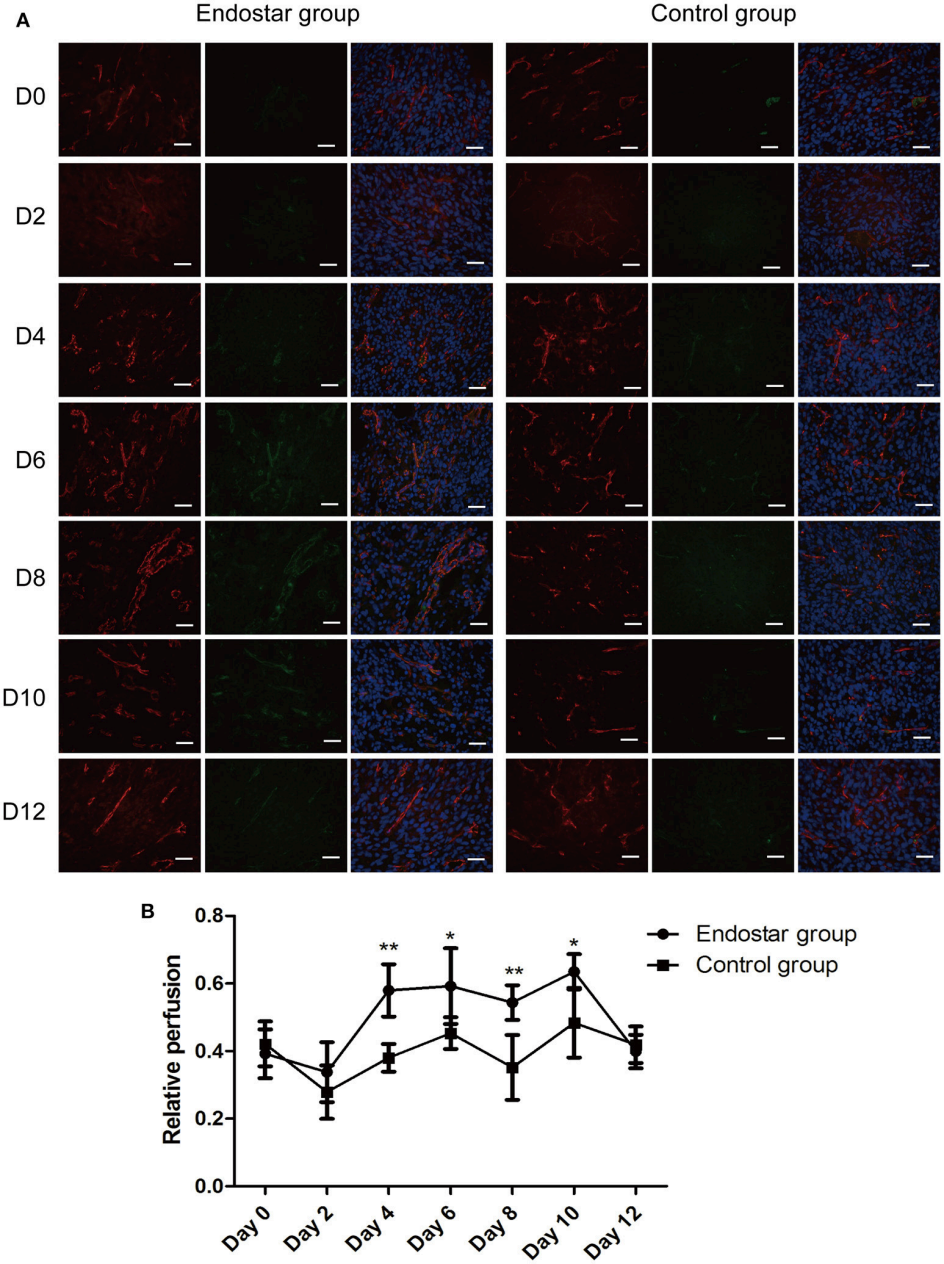
Evaluation of the vascular normalization window by histologic analysis of vascular perfusion. **(A)** Lectin perfusion (green) and CD31 (red) staining of tumor sections of the Endostar and control groups at different time points after treatment. **(B)** Changes and differences in relative perfusion (lectin+ area/CD31+ area) in the two groups at each time point (days 0, 2, 4, 6, 8, 10, and 12 after treatment; *P < 0.05; **P < 0.01). CD31, cluster of differentiation 31.

We further evaluated the vascular normalization window by performing histologic analysis of vascular perfusion, which is another feature of tumor vascular normalization. As shown in Figure 6A, the relative perfusion in tumors of the Endostar group improved markedly for 4 days after treatment and then decreased significantly after day 10. The Endostar group exhibited a significantly higher relative perfusion than the control group on days 4, 6, 8, and 10 after treatment. There was no significant difference in relative perfusion between the two groups on days 0, 2, and 12 (*P* < 0.05; Figure 6B). On the basis of the trends in variation and differences in the histologic findings of vascular perfusion between the two groups, we further confirmed that Endostar treatment induced the CT26 tumor vascular normalization window starting from day 4 after treatment and that this window lasted for 6 days. These results also revealed that, in both groups, the trends in variation of perfusion and pericyte coverage were similar to those of the IVIM-derived D^*^ and f values.

### Correlation between the IVIM-derived and histologic parameters of tumor vascular normalization

**Figure 7:**
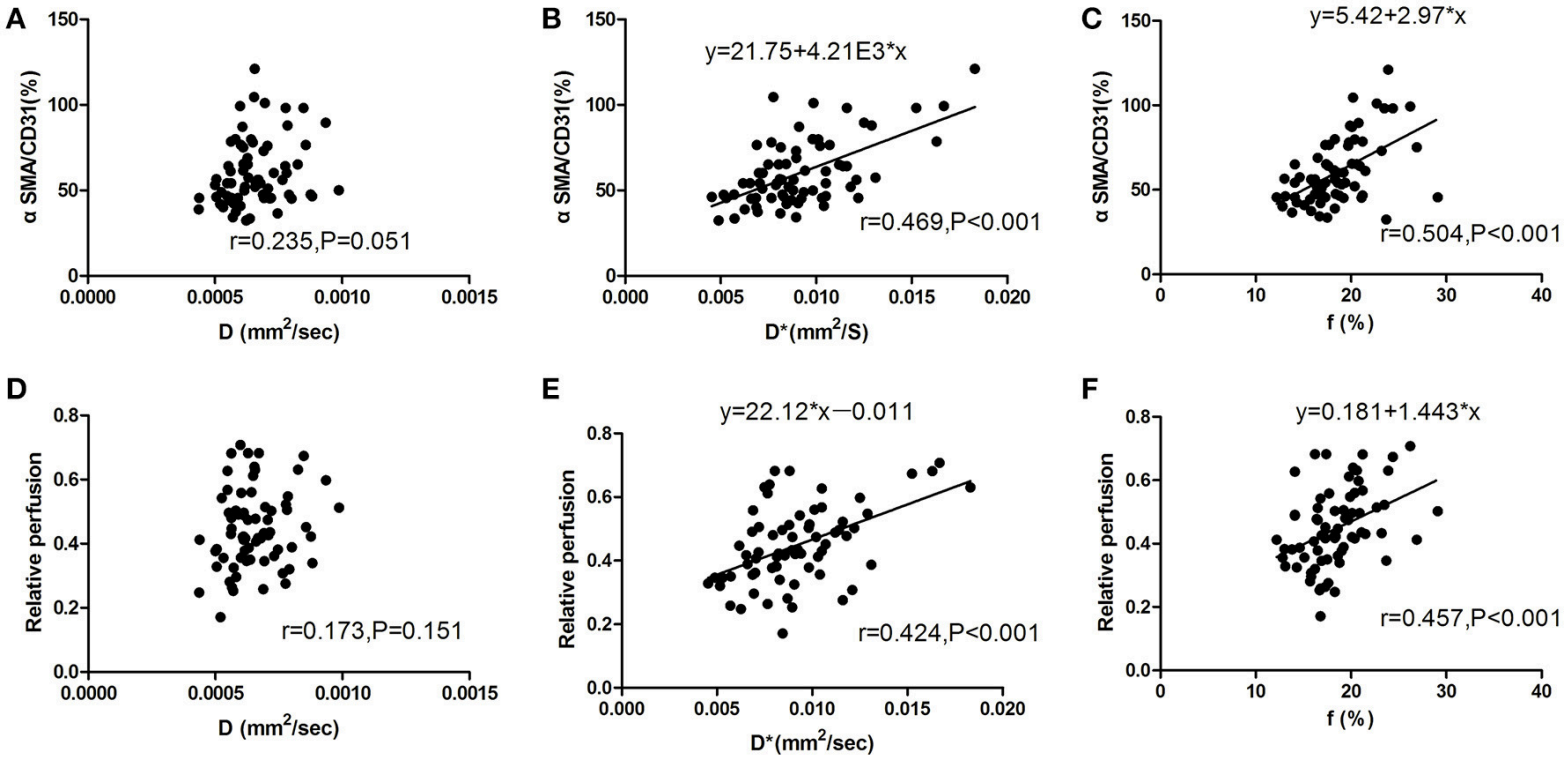
Correlation between IVIM-DWI MRI and histologic parameters for analysis of tumor vascular normalization. **(A)** There was no significant correlation between D value and pericyte coverage (*r* = 0.235; *P* = 0.051). **(B,C)** D*value (*r* = 0.469; *P* < 0.001) and f value (*r* = 0.504; *P* < 0.001) were significantly correlated with pericyte coverage during the process of tumor vascular normalization. **(D)** There was no significant correlation between D value and relative perfusion (*r* = 0.173; *P* = 0.151). **(E)** D*value and **(F)** f value were both positively correlated with relative perfusion (correlation coefficients, 0.424 and 0.457, respectively; *P* < 0.001). CD31, cluster of differentiation 31; SMA, smooth muscle actin; IVIM, intravoxel incoherent motion; DWI, diffusion-weighted imaging; MRI, magnetic resonance imaging.

The correlation between the IVIM-derived parameters and histopathologic features of tumor vascular normalization in colon cancer still remains unclear. Therefore, we calculated the correlation coefficients between IVIM-derived parameters and pericyte coverage or vascular perfusion. As shown in Figure 7, pericyte coverage exhibited a good correlation with both D^*^ value (*r* = 0.469; *P* < 0.001) and f value (*r* = 0.504; *P* < 0.001) in the CT26 tumor model. However, there was no significant correlation between D value and pericyte coverage (*r* = 0.235; *P* = 0.051). With regard to vascular perfusion, both D^*^ and f values were significantly correlated with relative perfusion (*r* = 0.424 and 0.457, respectively; *P* < 0.001, both), while D value exhibited no significant correlation with relative perfusion.

## Discussion

Tumor vascular normalization offers new opportunities in anti-angiogenic therapeutics for cancer, especially in combination with chemoradiotherapy. However, it still remains a challenge to monitor the process of vascular normalization and identify the window of vascular normalization in patients (23). Therefore, there is an urgent need to identify a novel detection method that offers the advantages of non-invasiveness, timeliness, and good diagnostic performance. In this study, we showed that IVIM DW-MRI can help monitor the process of vascular normalization. Using the findings of IVIM DW-MRI, we also identified the window in which Endostar induces vascular normalization. We further found a good correlation between the IVIM-derived and histologic parameters of the colon carcinoma-bearing mouse model. To our knowledge, this is the first study to monitor the process of vascular normalization by means of IVIM DW-MRI and identify the correlation between IVIM-derived and histologic parameters in Endostar-induced vascular normalization of colon cancer.

As reported in a previous study which evaluated tumor vascular normalization mainly on the basis of changes in vascular structure and function (24), improvement in pericyte coverage is indicative of more mature tumor blood vessels, and a greater extent of pericyte coverage will support endothelial integrity, stabilization, and maturation. Another feature of tumor vascular normalization is improvement in vascular function, such as increase in intratumoral perfusion and oxygenation and improvement in delivery of drugs (25). These phenomena of vascular normalization have been observed during treatment with anti-angiogenic factors, including Endostar (26, 27). Therefore, histologic findings showing an increase in intratumoral pericyte coverage and enhancement of vascular perfusion are considered as the gold standard for assessing the process of tumor vascular normalization (11). For assessing the vascular normalization window in this study, we first identified that the vascular normalization window started on day 4 and lasted up to 6 days after Endostar treatment, on the basis of IVIM DW-MRI findings. Our results revealed that the IVIM-derived f and D^*^ values exhibited similar tendencies as the histologic parameters, including pericyte coverage and vascular perfusion. Thus, these results revealed that IVIM-derived f and D^*^ values can reflect the changes in the vascular structure and function of colorectal cancer after Endostar treatment.

In order to consider further applications for IVIM DW-MRI, it is essential to identify the exact correlation between histologic characteristics and IVIM DW-MRI-derived parameters in different tissues. Recently, several papers have reported on the relationship between IVIM-derived and histologic parameters. In pancreatic ductal adenocarcinomas, f value has been reported to show an excellent correlation with microvessel density (MVD) and microvessel area (18). Another study has reported that f value is also positively correlated with MVD in orthotopic rat glioma models (28). Both D^*^ and f values have also been reported to be correlated with MVD in tumor specimens with vascular networks marked by anti-CD31 immunohistochemical staining (19). These results indicate that the D^*^ and f values of IVIM DW-MRI can be considered as non-invasive markers for tumor vascularity. Since the poor functional nature of neovessels will impact the IVIM-derived D^*^ values in tumors (29), we used α-SMA and CD31 as immunofluorescence markers for performing histologic assessment of tumor vascular structure in our study. This combination of markers can better reflect the complete structure of the vasculature than either one of the markers alone. Our results showed that, in the CT26 model, histologically determined pericyte coverage is well correlated with IVIM-derived D^*^ and f values but not with the D value. However, a previous study (30) has reported that IVIM-derived parameters are well correlated with DCE-MRI parameters as well as vessel maturity index in a bevacizumab-induced vessel normalization model of orthotopic rat glioma. In our study, we identified, for the first time, the Endostar-induced normalization window in a subcutaneous colon carcinoma model by using IVIM-DWI MRI. Our results indicating that D^*^ and f values are positively correlated with vessel pericyte coverage in tumors treated with anti-angiogenic factors are consistent with those of previous studies. Since immunofluorescence is more sensitive than immunohistochemical analysis for immunostaining within the vasculature (31), our study might have potentially calculated more accurate correlation coefficients between IVIM DW-MRI parameters and histologic characteristics of tumor vessel normalization than previous studies.

Furthermore, our results demonstrated that D^*^ and f values, but not D value, are also well correlated with histologically determined vascular perfusion parameters. Histologically determined MVD and pericyte coverage cannot reflect tumor perfusion. Furthermore, the pathological characteristics of tumor vessel normalization include not only vessel maturity but also intratumoral perfusion. Therefore, to determine accurate correlation coefficients between IVIM-derived parameters and tumor perfusion, we assessed intratumoral perfusion by intravenously injecting CT26 tumor-bearing mice with FITC-conjugated lectin. The good correlation of D^*^ and f values with histologically determined vascular perfusion parameters further confirmed that IVIM DW-MRI can serve as a novel method for determining the window of tumor vascular normalization. Indeed, this possibility was confirmed by the interesting results obtained by using IVIM DW-MRI for assessment of tumor vascular normalization in this study. However, we have to admit that the clinical efficacy of IVIM DW-MRI in monitoring tumor vascular normalization still needs further research. Additionally, diagnostic standards for monitoring tumor vascular normalization using IVIM DW-MRI in patients should be established in the future.

There are several limitations to our study. First, although we determined the correlation of IVIM-MRI parameters with pericyte coverage and relative perfusion in this study, the relationship between IVIM-MRI parameters and other features of tumor vascular normalization, such as oxygenation and delivery of drugs, still needs to be confirmed. Second, the tumor vascular normalization window is dependent on tumor type, treatment schedule, and type of VEGF signaling inhibitor used. However, since our study only focused on Endostar treatment in a colon cancer model, whether IVIM-MRI is effective for monitoring vascular normalization in other types of cancers or drug-treatment regimens is not clear, and this aspect should be evaluated in a comparative study.

In conclusion, IVIM DW-MRI appears to be a novel approach for obtaining information on the process of tumor vascular normalization, as confirmed by the good correlation between the present imaging and histologic findings. Our results showed that IVIM-derived D^*^ and f values are well correlated with histologically determined vascular structure and perfusion parameters in Endostar-induced vascular normalization of colon tumors. Therefore, IVIM DW-MRI has the potential to serve as a non-invasive approach for monitoring Endostar-induced tumor vascular normalization.

## Author contributions

All authors listed have made a substantial, direct and intellectual contribution to the work, and approved it for publication.

### Conflict of interest statement

The authors declare that the research was conducted in the absence of any commercial or financial relationships that could be construed as a potential conflict of interest.
